# Efficacy and safety of chloral hydrate sedation in infants for pulmonary function tests

**DOI:** 10.1016/j.rppede.2016.06.001

**Published:** 2016

**Authors:** Gustavo Falbo Wandalsen, Fernanda de Cordoba Lanza, Márcia Cristina Pires Nogueira, Dirceu Solé

**Affiliations:** a Departamento de Pediatria, Escola Paulista de Medicina, Universidade Federal de São Paulo (Unifesp), São Paulo, SP, Brazil; b Universidade Nove de Julho, São Paulo, SP, Brazil

**Keywords:** Chloral hydrate, Hypnotics and sedatives, Infant

## Abstract

**Objective:**

To describe the efficacy and safety of chloral hydrate sedation in infants for pulmonary function tests.

**Methods:**

All sedation attempts for pulmonary function tests in infants carried out between June 2007 and August 2014 were evaluated. Obstructive sleep apnea and heart disease were contraindications to the exams. Anthropometric data, exam indication, used dose, outcomes of sedation and clinical events were recorded and described.

**Results:**

The sedation attempts in 277 infants (165 boys) with a median age of 51.5 weeks of life (14-182 weeks) were evaluated. The main indication for the tests was recurrent wheezing (56%) and the chloral hydrate dose ranged from 50 to 80mg/kg (orally). Eighteen (6.5%) infants had some type of clinical complication, with the most frequent being cough and/or airway secretion (1.8%); respiratory distress (1.4%) and vomiting (1.1%). A preterm infant had bradycardia for approximately 15 minutes, which was responsive to tactile stimulation. All observed adverse effects were transient and there was no need for resuscitation or use of injectable medications.

**Conclusions:**

The data demonstrated that chloral hydrate at the employed doses is a safe and effective medicament for sedation during short procedures in infants, such as pulmonary function tests. Because of the possibility of severe adverse events, recommendations on doses and contraindications should be strictly followed and infants should be monitored by trained staff.

## Resumo

**Objetivo:**

Descrever a eficácia e a segurança do hidrato de cloral na sedação de lactentes para testes de função pulmonar.

**Métodos:**

Foram avaliadas retrospectivamente todas as tentativas de sedação para exames de função pulmonar em lactentes feitas entre junho 2007 e agosto 2014. Apneia obstrutiva do sono e cardiopatia foram contraindicações para os exames. Dados antropométricos, indicação do exame, dose empregada, desfechos da sedação e intercorrências clínicas foram registrados e descritos.

**Resultados:**

Avaliaram-se as tentativas de sedação de 277 lactentes (165 meninos) com mediana de 51,5 semanas de vida (14-182). A principal indicação para os testes foi sibilância recorrente (56%) e a dose de hidrato de cloral usada variou entre 50-80mg/kg (via oral). Dezoito (6,5%) lactentes apresentaram algum tipo de intercorrência clínica e foram as mais frequentes: tosse e/ou secreção na via aérea (1,8%); desconforto respiratório alto (1,4%) e vômitos (1,1%). Um lactente prematuro apresentou bradicardia por cerca de 15 minutos, responsiva a estimulação tátil. Todos os efeitos adversos observados foram transitórios e não houve necessidade de manobras de reanimação e uso de medicações injetáveis.

**Conclusões:**

Os dados demonstraram que o hidrato de cloral, nas doses empregadas, é um medicamento seguro e eficaz para a sedação de lactentes em procedimentos de curta duração, como os testes de função pulmonar. Devido à possibilidade de eventos adversos graves, as recomendações referentes à dosagem e contraindicações devem ser seguidas de forma rígida e os lactentes devem ser monitorados por equipe treinada.

## Introduction

Chloral hydrate (CH) is a hypnotic sedative drug widely used in recent decades to sedate children. Although its exact mechanism of action remains uncertain, the CH is metabolized to trichloroethanol, the active metabolite responsible for the hypnotic effects. The half-life of this metabolite is 8-12h in preschoolers, but can be up to four times longer in newborns and preterm infants.^1^^,^ 2

CH is the sedative of choice for pulmonary function tests in infants and has been used in several laboratories for more than 25 years.^1^^,^ 3 It is the drug of choice due to several factors. It is administered orally, does not require venipuncture and induces adequate sedation degree and duration for pulmonary function assessment procedures to be carried out.^1^ Additionally, the available reference equations for pulmonary function parameters were obtained after sedation with CH and use of other sedatives could hinder comparisons and induce biases.^3^ In a recent survey carried out at 148 pulmonary function laboratories in infants worldwide, 79% of them used CH as a sedative for exams.^4^

The use of CH for sedation in young children, however, is not a consensus and severe adverse events, including deaths, have been reported.^2^^,^ 5 Recently, the Brazilian National Health Surveillance Agency (ANVISA) has banned the sale of CH in the country for lack of evidence of its efficacy and safety. The aim of this article is to describe the experience of the pulmonary function laboratory in infants treated at the Discipline of Allergy, Clinical Immunology and Rheumatology of the Department of Pediatrics of Escola Paulista de Medicina (Unifesp) regarding the use of CH in necessary sedation for pulmonary function tests.

## Method

This is a retrospective case series, which evaluated all sedation attempts for pulmonary function tests in infants between June 2007 and August 2014. The pulmonary function tests were carried out in infants weighing ≥4kg and no history of respiratory infection in the previous two weeks. On the day of the pulmonary function test, the infants that came to the service had fasted for at least 3h and received an oral dose of chloral hydrate after clinical evaluation. All tests were performed with the infant in the supine position with slight neck extension and tests were carried out with continuous heart rate and oxygen saturation monitoring. A physician and a physical therapist trained in emergency care were present in all examinations and resuscitation equipment was available during all tests.

Lung volumes and forced expiratory flow were measured using specific equipment (Infant Pulmonary Lab, Collins-nSpire, USA), according to existing recommendations.^6^^,^ 7 In brief, lung volume was measured during the infants' spontaneous breathing movements; then, the airway was occluded for a few seconds, at some moments during breathing. Forced expiratory flows were obtained using the rapid thoracoabdominal compression technique with high lung volumes. Flow-volume curves were obtained by compression of an inflatable jacket placed around the infant's chest and abdomen after inspiration with positive pressure (30cm/H_2_O). Chest and abdomen compression was maintained until the end of expiration was visually identified or for a maximum period of four seconds. Several curves were obtained with increasing thoracoabdominal compression pressure, until there was no increase in forced flow and volume values.

To evaluate the safety and efficacy of CH in this study, anthropometric data, test indication, CH dose employed (per kg of bodyweight) and sedation outcome were recorded, as well as successful sedation and pulmonary function assessment. In cases of clinical complications, the nature of these complications and required procedures were recorded.

All tests were performed after obtaining written consent from parents and/or guardians and after approval of the Institutional Review Board of Universidade Federal de São Paulo (Unifesp).

## Results

A total of 277 infants were assessed (165 males), with a median age of 51.5 weeks (range 14-182). The indications for the tests were: prematurity and/or low birth weight in 74 (27%), recurrent wheezing and/or lung disease in 156 (56%), sickle-cell anemia in 13 (5%) and others in 34 (12%) infants.

CH dose ranged from 50 to 80mg/kg (orally), with a median of 70mg/kg. This dose usually induces sleep quickly (in approximately 20min) and maintains it for approximately 50min. Of the attempted assessments, it was not possible to perform the pulmonary function test in 32 (12%) cases: 18 (6.5%) due to problems in sedation (9 infants did not sleep and 9 awakened too soon), 10 (4%) due to clinical problems and 4 (1%) due to technical difficulties. Clinical problems that prevented the assessment from being performed were cough (2 infants); respiratory distress (1); vomiting (3); presence of airway secretions (1); decrease in peripheral oxygen saturation (SpO_2_) (1); bradycardia (1) and upper airway obstruction (1).

In the 245 pulmonary function tests performed, clinical events were observed during the examination in eight infants (3%). Thus, of the 277 sedation attempts, 18 (6.5%) infants showed some type of clinical complication after CH administration. The observed adverse events are shown in Table 1.

**Table 1:** Adverse events observed after administration of chloral hydrate in 277 infants.

	Number	Percentage
Cough and/or airway secretion	5	1.8
Upper respiratory distress	4	1.4
Vomiting	3	1.1
Decrease in oxygen saturation	2	0.7
Abdominal distension	2	0.7
Prolonged hiccups	1	0.4
Bradycardia	1	0.4

The actions required in these cases were upper airway aspiration, cervical repositioning and oxygen supplementation. The child that had bradycardia was a preterm infant, born at 27 weeks of gestation weighing 770g, and a chronological age of 11 months at the time of the examination. After sedation with 75mg/kg of CH, the heart rate decreased for about 15min, not lower than 65bpm, being responsive to tactile stimulation.

All adverse events observed during the 277 sedation attempts were transient, with spontaneous improvement after the end of CH action. There were no cases that required ventilation and resuscitation maneuvers, use of injectable medications or hospitalization.

## Discussion

CH-induced sedation is considered of moderate intensity and, therefore, it is recommended that it be performed under the supervision of physicians and health professionals trained in life support, with available resuscitation equipment and infant monitoring.

It is known that the CH can reduce upper airway muscle tone and increase the chance of collapse in infants with some degree of upper airway obstruction, such as those with pharyngeal and palatine tonsil hypertrophy, obstructive sleep apnea and craniofacial abnormalities.^1^ For this reason, such conditions contraindicate pulmonary function test performance in our laboratory. Similarly, examinations are not carried out in infants with heart disease due to the potential cardiac depression and induction of conduction disorders caused by CH, which causes arrhythmias.^1^

Some comments about our data are important. Overall, our findings reinforce data from other services and demonstrate the safety and efficacy of CH as a sedative to be used for procedures in infants.^8^^-^ 10

The CH dose used by us (50-80mg/kg) was similar to that employed by other investigators^4^ and sedation failure was observed in only 6.5% of the attempts. Higher doses of the sedative and/or repeated doses could reduce the number of failures, but are associated with increased risk of adverse events.^1^ West et al. evaluated the efficacy and safety of CH in infants and preschoolers for ophthalmic procedures.^9^ Using a dose of 80mg/kg of CH, they observed inadequate sedation in 7.2% of children. An additional dose of up to 40mg/kg was administered in cases of failure, with sedation failure being reduced to 3.3%.^9^

In our service, we observed an incidence of 6.5% of adverse events. The observed adverse events and their frequency were similar to those described by other authors, that is: paradoxical reactions (1.3%); decrease in SpO_2_ (1%); vomiting (0.5%) and bradycardia (0.1%).^9^ The report of another large case series, by Avlonitou et al. compiled adverse events after sedation with CH for hearing tests in 1903 children up to 14 years of age (568 children under six months of age).^8^ In this study, they observed hyperactivity in 8% of cases, vomiting in 11%, mild respiratory discomfort 0.5% and apnea in 0.2% of the cases.^8^

Prematurity and younger age are factors known to be associated with increased risk of adverse events after sedation with CH.^10^ These two factors were present in the case of the infant who had bradycardia in our laboratory. As observed by us, other authors have reported that cases of bradycardia induced by CH tend to be responsive to physical stimuli, with good evolution.^9^^,^ 10

The safety of CH (80mg/kg) has also been studied in a group of 1095 children (aged between one month and three years) sedated for echocardiograms.^11^ In this risk group, consisting mostly of patients with heart disease, 10.8% had some adverse event, such as decrease in SpO_2_ (5.9%), hypercapnia (6.6%), airway obstruction (1.4%), apnea (0.3%), hypotension (0.4%) and vomiting (0.4%). Of the assessed children, 24% showed a decrease in heart rate ≥20%, but only 1.4% decreased heart rate below the normal range for age.^11^ Major interventions such as face-mask ventilation and volume expansion were necessary in five cases (<0.5%), with tracheal intubation in one of them.^11^

A review of adverse event reports associated with sedation in North-American children identified 95 severe cases reported by the end of the 1990s.^5^ Of these, 20 were related to the use of CH, with 13 cases leading to death or permanent neurological damage. Among these more severe cases, CH was administered alone in seven patients and in combination with other drugs in six.^5^ CH overdose was identified in four cases and nine had pre-existing clinical problems, such as tracheomalacia, tracheostomy, genetic syndromes, congenital heart defects and cerebral palsy.^5^

Despite the existence of these severe adverse event reports, CH has been administered for pulmonary function tests in a large number of studies involving thousands of infants with respiratory problems, with a low incidence of adverse events and no report of death or permanent sequelae.^1^

Several other sedative drug options for infants are available, each showing specific advantages and disadvantages. Midazolam is a short-duration benzodiazepine that is widely used in clinical practice, especially intravenously. In addition to the need for venous access, midazolam can also cause respiratory depression and hypotension.^12^ The main advantage of midazolam is the availability of an antagonist able to reverse its effects.^12^ More recently, the intranasal formulation of midazolam has been developed, which is more convenient for outpatient procedures, but with less sedative action. Dexmedetomidine is a selective α2-adrenergic agonist capable of inducing sedation and slight analgesia, used in several diagnostic procedures.^13^ It is preferably administered intravenously, with a small failure rate, and its most common adverse effects are bradycardia and blood pressure changes.^13^^,^ 14 The effect of dexmedetomidine on the respiratory system is small and, unlike CH, the sedative seems to be safe in children with upper airway obstructive disorders, but studies in young children are still limited.^14^

In conclusion, the data obtained in our service are similar to those observed by other authors and demonstrate that CH, at the assessed doses, is a safe and effective drug for infant sedation during short procedures, such as pulmonary function tests. Due to the possibility of severe adverse events, dose recommendations and contraindications to the use of CH should be strictly followed. Sedation with CH should always be carried out by trained staff, under medical supervision and continuous monitoring of infants. Special attention should be given to infants belonging to risk groups, such as preterm infants.
